# Validation of motion tracking as tool for observational toothbrushing studies

**DOI:** 10.1371/journal.pone.0244678

**Published:** 2020-12-30

**Authors:** Carolina Ganss, Patrick Klein, Katja Giese-Kraft, Michael Meyners

**Affiliations:** 1 Department of Conservative and Preventive Dentistry, Dental Clinic of the Justus-Liebig-University Giessen, Giessen, Germany; 2 Procter & Gamble Service GmbH, Kronberg, Germany; University of Zurich, SWITZERLAND

## Abstract

Video observation (VO) is an established tool for observing toothbrushing behaviour, however, it is a subjective method requiring thorough calibration and training, and the toothbrush position is not always clearly visible. As automated tracking of motions may overcome these disadvantages, the study aimed to compare observational data of habitual toothbrushing as well as of post-instruction toothbrushing obtained from motion tracking (MT) to observational data obtained from VO. One-hundred-three subjects (37.4±14.7 years) were included and brushed their teeth with a manual (MB; n = 51) or a powered toothbrush (PB; n = 52) while being simultaneously video-filmed and tracked. Forty-six subjects were then instructed how to brush their teeth systematically and were filmed/tracked for a second time. Videos were analysed with INTERACT (Mangold, Germany); parameters of interest were toothbrush position, brushing time, changes between areas (events) and the Toothbrushing Systematic Index (TSI). Overall, the median proportion (min; max) of identically classified toothbrush positions (both sextant/surface correct) in a brushing session was 87.8% (50.0; 96.9), which was slightly higher for MB compared to PB (90.3 (50.0; 96.9) vs 86.5 (63.7; 96.5) resp.; p = 0.005). The number of events obtained from MT was higher than from VO (p < 0.001) with a moderate to high correlation between them (MB: *ρ* = 0.52, p < 0.001; PB: *ρ* = 0.87; p < 0.001). After instruction, both methods revealed a significant increase of the TSI regardless of the toothbrush type (p < 0.001 each). Motion tracking is a suitable tool for observing toothbrushing behaviour, is able to measure improvements after instruction, and can be used with both manual and powered toothbrushes.

## Introduction

Removing dental plaque in a sufficient and effective way is a prerequisite for maintaining oral health. To achieve this, various types of manual and powered toothbrushes are available. Many studies have evaluated the efficacy of toothbrush types and toothbrushing techniques mainly using plaque or gingivitis indices as target parameter [1–5]. Though these parameters are of clinical relevance, they are surrogates with respect to the question how toothbrushing was performed.

Getting insights into motion habits during toothbrushing, however, may help to improve instruction strategies. To this end, video observation (VO) is a method allowing for analysing brushing behaviour in an elaborated way. VO studies indeed revealed that patients predominantly brush on vestibular surfaces, frequently switch between the left and right side of the dentition, and often neglect oral surfaces almost completely [6–11].

Though video observations brought valuable insights into patient’s oral hygiene behaviour, some shortcomings are obvious. It is a subjective procedure requiring thorough calibration and training, it is time consuming, and the position of the toothbrush is not always clearly visible.

Meanwhile, novel approaches like robotics, artificial intelligence and machine learning receive more and more attention in dentistry [12]; thus, an alternative to personal observation is capturing motions by wearable sensors like accelerometers or gyroscopes, or by infra-red/ultrasound cameras (e.g. [13, 14]). In toothbrushing observation studies, however, motion tracking (MT) has only been scarcely used so far.

In one study [15], toothbrushing gestures were recognised with a smart watch containing an accelerometer, a gyroscope, a gravity sensor, a magnetic sensor, and a microphone. The position of the toothbrush was identified by installing magnets on the toothbrush interfering with the smart watch. Several sets of data of defined toothbrushing were used to train a machine learning model. The authors state that toothbrushing surface precision was about 86% and that incorrect toothbrushing techniques are detected at high rates.

Another study [16] used an optical 3-D motion analyser. Six charge-coupled device (CCD) cameras recognised the motion of light-reflective balls attached to various areas of the body and to the toothbrush and the gestures were visualised on a computer display. The system tracked the motion of shoulder, elbow and wrist as well as joint angles when test subjects performed defined brushing procedures. The brushing frequency was recorded but not the position of the toothbrush relative to the dental arch. In contrast to these relatively complex tracking systems, a further study [17] suggested mounting a 3D coloured target at the end of the toothbrush enabling to track and analyse its motion in combination with a tablet and a facial localisation software. The device has been used in a small group of children, but so far, only preliminary data were presented.

As the tracking of motions (MT) during toothbrushing has not been systematically evaluated, the present study aims at comparing data and results from video observations (VO) to those from motion tracking. Parameters of interest were the position of the toothbrush per time point, the overall brushing time, the brushing time on areas of the dentition, the number of changes between areas of the dentition (events) and the degree of toothbrushing systematics.

The study consists of two parts: the first part of the study is the observation of a single habitual toothbrushing event. The second part is an instruction experiment aiming at investigating whether the motion tracking system is able to demonstrate changes of toothbrushing behaviour following a video instruction compared to changes of toothbrushing behaviour shown via video analysis. Parameters of interest were baseline to post instruction changes of the parameters mentioned above. Both manual (MB) and powered (PB) toothbrushes were investigated in the two parts of the study.

### Participants, materials and methods

The study took place at the Research Unit of the Procter & Gamble Service GmbH group, Kronberg, Germany. It was performed according to the guidelines of Good Clinical Practice and the Declaration of Helsinki and was approved by the Ethics Committee of the Justus-Liebig-University, Giessen, Germany (Doc. No 158/18).

The study group consisted of 103 subjects (35 male, 68 female) with a mean (±SD) age of 37.4±14.7 years enrolled from regional dental practices via oral and written announcements.

The study was conducted between October 30, 2018 and December 12, 2018. One hundred sixteen subjects were screened for this study, 8 of which were screening failures based on the following inclusion and exclusion criteria. Inclusion criteria were: written informed consent, at least 18 years of age, full dentition except for extraction for orthodontic reasons and except missing teeth replaced by bridgework or implants, good general health based on their medical history, agreement to return for their scheduled visit and to follow all study procedures. Exclusion criteria were: multiple severe carious lesions, active treatment for periodontitis, fixed orthodontic appliances (retainer permitted), or removable denture prosthesis, any disease or conditions that could be expected to interfere with examination procedures or with the subject safely completing the study (including allergies to imprint material).

One hundred eight subjects were enrolled in the study. One subject dropped after screening due to dental restoration problems, which were deemed unrelated to the intervention as it occurred prior to first visit. Four individuals were erroneously enrolled as they were employees of the sponsor. To avoid any potential bias, these subjects were excluded from the analysis.

### Study procedures

The study was a non-disguised observational study of toothbrushing behaviour with either a manual (Oral-B Indicator 35 soft, type OM010, Newbridge, Ireland) or a powered toothbrush (Oral-B Genius, type 3765, with a Cross Action Power brushhead, Marktheidenfeld, Germany). The first part investigated habitual toothbrushing behaviour and the second part brushing behaviour after instruction.

At the first visit, informed consent was obtained, and subjects were screened for inclusion/exclusion criteria. After inclusion in the study, an imprint of the upper and lower dentition was taken (Futar-D, Kettenbach, Eschenburg, Germany). At the next visit, subjects received either the manual (n = 51) or the powered toothbrush (n = 52) depending on their preferred home use toothbrush type. Subjects were positioned in the centre of the MT cameras and in front of a black box with a semi-transparent mirror and a video camera behind. They were asked to brush their teeth as normally, but to look at the mirror throughout and to show their teeth as much as possible while brushing. The brushing was recorded by the MT system and at the same time video filmed. Subjects brushed without toothpaste and without time restriction.

At the same visit, subjects were asked whether they were willing to participate also in the second part of the study. Forty-six subjects (manual brush n = 22; powered brush n = 24) were included and all received the same standardised video instruction how to brush in a systematic way. The video was specifically produced for the study and presented the toothbrushing systematics published by Rateitschak [18]. The video (118 s) showed the opened mouth of a fully dentate person and a dentist demonstrated the brushing sequence by moving a dental probe along the dental arcs. It started on oral surfaces on the right side of the lower jaw, continued to the anterior and to the contra lateral posterior area, then to the oral surfaces of the upper jaw moving from posterior to anterior and to the contra lateral posterior sites. Then, the probe was moved to the vestibular sites of the posterior area of the right upper jaw, continued to the anterior and posterior areas until ending at the posterior area of the right lower jaw. Finally, the occlusal surfaces were addressed. This was synchronously explained by spoken words. After watching the instruction video, subjects were asked to brush their teeth according to the instruction. Brushing was again recorded by VO and MT.

### Video observation and motion tracking procedures

VO was performed as described earlier [11], but using two cameras (Sony FDR-AX33). One camera was the main camera for video recording; the other was an adjunct camera providing additional views in cases where the toothbrush position was not clearly visible from the main camera recording. Both were synchronised with the MT system.

Videos were analysed using the software INTERACT (Mangold International GmbH, Amstorf, Germany) and were coded exhaustively, meaning that the whole observation session was coded for continuous timed-event behaviour sampling [19, 20]. Video analysis parameters were based on the studies from Macgregor and Rugg-Gunn [7, 8] and on previously performed video observation studies of our group [11, 21, 22]. For analysis, the dentition was divided into sextants (01–06) and surfaces (occlusal/incisal, vestibular and oral coded as 01, 02 and 03 resp.), resulting in 18 areas. The parameters of interest were brushing time and the sextant/surface where the toothbrush acted. From these parameters, the brushing time for each area and the number of events was calculated. To quantify the degree of toothbrushing systematics, the Toothbrushing Systematics Index (TSI) was calculated [21].

The observer (PK) was carefully trained and was calibrated using triplicate coding of 10 randomly selected videos analysed by an experienced observer from earlier studies with a manual (n = 5; [11]) and a powered toothbrush (n = 5; [22]). Kappa coefficients for inter- and intra-rater agreement were determined by INTERACT. In addition, the percentage of matching codes from the respective two analyses was calculated. The inter-rater agreement was calculated prior to the video analysis. The intra-rater agreement was determined prior to analysis (intra-rater agreement 1) and at mid-term of the VO analysis (intra-rater agreement 2). Respective data are presented in Table 1.

**Table 1:** Kappa coefficients and percentage of matching codes for areas (median (min; max)).

	Percentage of matching codes	Kappa coefficients
Inter-rater-agreement	88.8 (71.5; 98.9)	0.80 (surface)
		0.86 (sextant)
Intra-rater-agreement 1	88.2 (73.6; 97.1)	0.92 (surface)
		0.91 (sextant)
Intra-rater-agreement 2	88.3 (76.3; 97.1)	0.84 (surface)
		0.84 (sextant)

The motion tracking system consisted of eight infra-red cameras (Flir Grasshopper3 GS3-U3-23S6M with applied infra-red bandpass filter) which recorded signals from infra-red transmitters (tracker, weight 86 g, width 7 cm, height 10 cm) mounted on the toothbrushes and on a headgear. The cameras were positioned in a semi-circular arrangement in order to allow recording signals from the toothbrushes and from the headgear in an optimal way. The headgear allows participants to move freely within the allocated space; by associating position of the toothbrush relative to the headgear, the positional data remains unaffected from head or body movements. To teach the position of the tracker relative to the centre of the toothbrush head and to determine the position of the teeth in the dental arches, a teaching probe was used (short bar, also equipped with a tracker to allow teaching the position of individual teeth). The positions of the teeth were determined on the imprints by touching the midpoints of the occlusal and incisal surfaces of all teeth as well as the proximal points of the lower and upper central incisors with the probe. Teaching of the imprints and the toothbrushes was performed using standardised holders on a teaching desk. Teaching of the upper jaw relative to the headgear tracker was done by repositioning the imprint connected with a tracker into the mouth. The positions of the two trackers (headgear and imprint) relative to each other were then taught to the system. Next, the imprint of the lower jaw was repositioned and the jaw was taught in closed as well as in maximally open position. All procedures followed a standard operation procedure. The MT system components and procedures are described in detail in [23].

To estimate the effect of the tracker mounted on the toothbrush on brushing performance, 12 of the study subjects (6 with manual and 6 with powered brushes) brushed again without the tracker after brushing with the tracker in place for the main study.

There was no significant difference between the time spent in the different sextants when brushing with or without tracker. The only exception was sextant 5 (manual brush; p = 0.025), but the mean difference was 2.0 s which is of minor relevance.

The mean difference (with tracker—without tracker) of the mean time spent in the sextants ranged between 1.0 and 4.6 s for manual brushing and between -1.8 and 3.2 s for powered brushing. Despite the rather small sample sizes, 90% confidence intervals (related to equivalence tests at level 5%) were well within [-8.0, 8.0] except for sextant 1 for manual brushing for which a large variation was observed and the upper limit slightly exceeded 10 s.

### Statistics

#### Sample size calculation

As only very few observational studies on toothbrushing behaviour have been published, the planned sample size is based on earlier studies of our working group [11, 21, 22]. These studies have shown that a group size of around 100 is suitable to represent a wide range of behaviour related to toothbrushing and to allow for statistically sound comparisons of parameters of interest. An intervention study [10] aiming at comparing different types of oral hygiene instruction with respect to adopting the new technique has shown that a group size of 30 in each of three study arms produced sufficient data for statistical analysis. This sample size included potential dropouts, which was not a concern in the present study as all evaluations took place in the same session, so a sample size of 20–25 was deemed sufficient.

#### Data processing and statistics

Data from VO as well as from MT were pre-processed to convert them into a common format with relevant information (type of toothbrush, frame / time, area, synchronization time, begin and end of brushing session). Thereby, angles of 20° and 13° in MT were used as thresholds between occlusal and incisal versus oral/vestibular brushing. MT data were recorded with a sampling rate of 100 Hz while videos were recorded with 50 frames per second. Duration based data were aggregated into half (0.5) seconds for both systems, since it was assumed that brushing events typically need to last more than 0.5 s to be coded as a separate event by VO. For each interval of 0.5 s, the MT data were compared against VO data to determine whether the information matches. When MT information was inconsistent, the majority vote was taken, i.e. the area that was recorded most frequently within the respective time interval. In case of identical frequencies for different areas, the one occurring first among those with highest count was used; based on the interval-summarized data after these corrections, allocated areas, sextants and surfaces of the toothbrush head from VO and MT were cross-tabulated and the confusion matrix assessed.

The proportion of matching codes from both methods was determined. The number of events as well as the brushing duration within areas was determined. When subjects brushed the vestibular surfaces of sextants 01–06, 02–05 or 03–04 at the same time (occurring when subjects moved the toothbrush across sextants while jaws were closed), duration was evenly distributed across the two respective sextants. TSI scores [21] as well as its two sub-measures consistency (*C*) and isochronicity (*I*) were based on vestibular and oral surfaces (12 areas) only, ignoring occlusal/incisal brushing. Consistency is determined as

$$C=max\left(0;\;(1-\frac{b}{x})∙\frac{i}{n}\right)$$

with *b* the number of changes between areas, *x* the total brushing duration in seconds, *i* the number of reached areas, and *n* the total number of reachable areas (i.e. 12 here). With the same notation and with *d*_*i*_ the brushing duration in seconds in area *i*, isochronicity is determined as

$$I=1-\frac{n}{2(n-1)}\sum_{i=1}^{n}\left|\frac{d_{i}}{x}-\frac{1}{n}\right|$$

Both measures take values in [0, 1], with larger values indicating more systematic brushing. The overall TSI score is defined as the sum of *C* and *I* [21].

Analyses were performed using R 3.6.3 [24]. Due to potential violations of the normality assumption, non-parametric procedures were generally used, and data are given as median (min; max). Independent observations were compared using the Mann-Whitney-*U* test, while for comparisons of paired (dependent) data, the Wilcoxon signed-rank test was employed. For correlations, Spearman rank correlation coefficient *ρ* was used. The level of significance was set at 0.05; p values from multiple comparisons (comparisons of brushing duration obtained from VO and MT in the 18 areas) were adjusted according to the procedure of Bonferroni-Holm. To identify any potential patterns in the mismatches, a heat map of the confusion matrix was created, where darker shades of grey indicate higher proportions of mismatches between the respective segments. The diagonal (representing the matches) was omitted from the calculations and is displayed in white, as the number of matches is in a different order of magnitude than that of mismatches and would hence obscure differences in the latter numbers. The total number of mismatches, if any, was added to every cell of the graph. Confusion between non-adjacent areas like, e.g., opposite sides of the mouth or vestibular versus oral surface, are shaded in red to identify the most problematic potential confusions with regard to consistency between the methods.

## Results

The data from this study is made available elsewhere [23] and contains the processed data with area information for every subject, session and time point that has subsequently been used for the analyses reported in the following.

### Correctly classified areas

**Fig 1:**
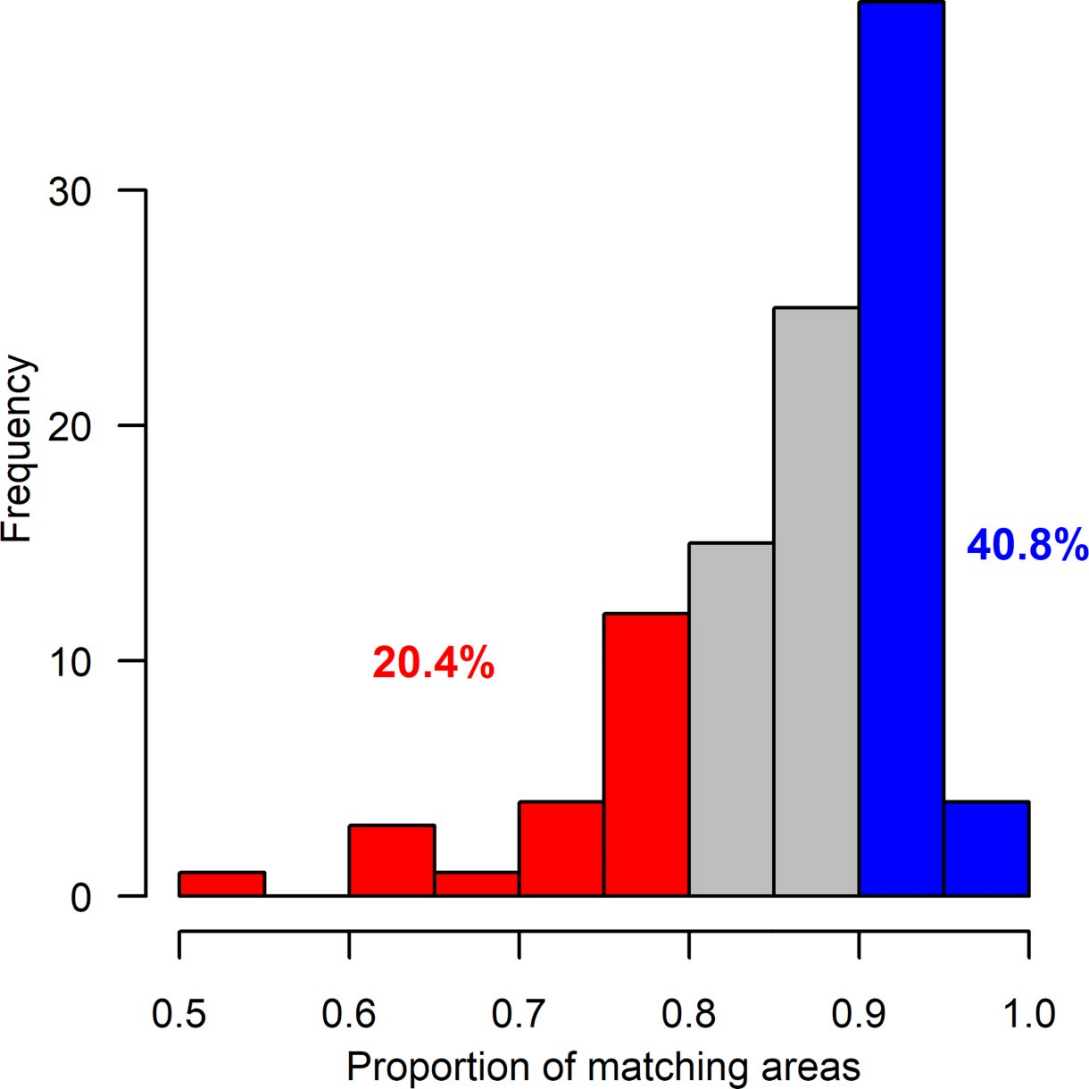
Histogram of the proportion of matching areas from the first part of the study. Colours are used to guide interpretation by highlighting proportions of matching areas above 90% and below 80% along with the observed relative frequencies.

For all cases from the first part of the study (i.e. before instructions, n = 103) the median proportion of matching areas (both sextant and surface identically classified by VO and MT) from the two methods was 87.8% (min = 50.0; max = 96.9); 79.6% of the cases had ≥80% matches and 40.8% of the cases had ≥90% matches. Fig 1 displays the histogram.

This proportion was slightly higher for manual (n = 51) compared to powered (n = 52) brushing (90.3 (50.0; 96.9) vs 86.5 (63.7; 96.5); p = 0.005).

Similar was true for baseline (87.7 (64.9; 96.9) versus instruction 83.8 (59.1; 94.8)) analyses based on those subjects that took part in both parts (n = 46, p = 0.041)

**Fig 2:**
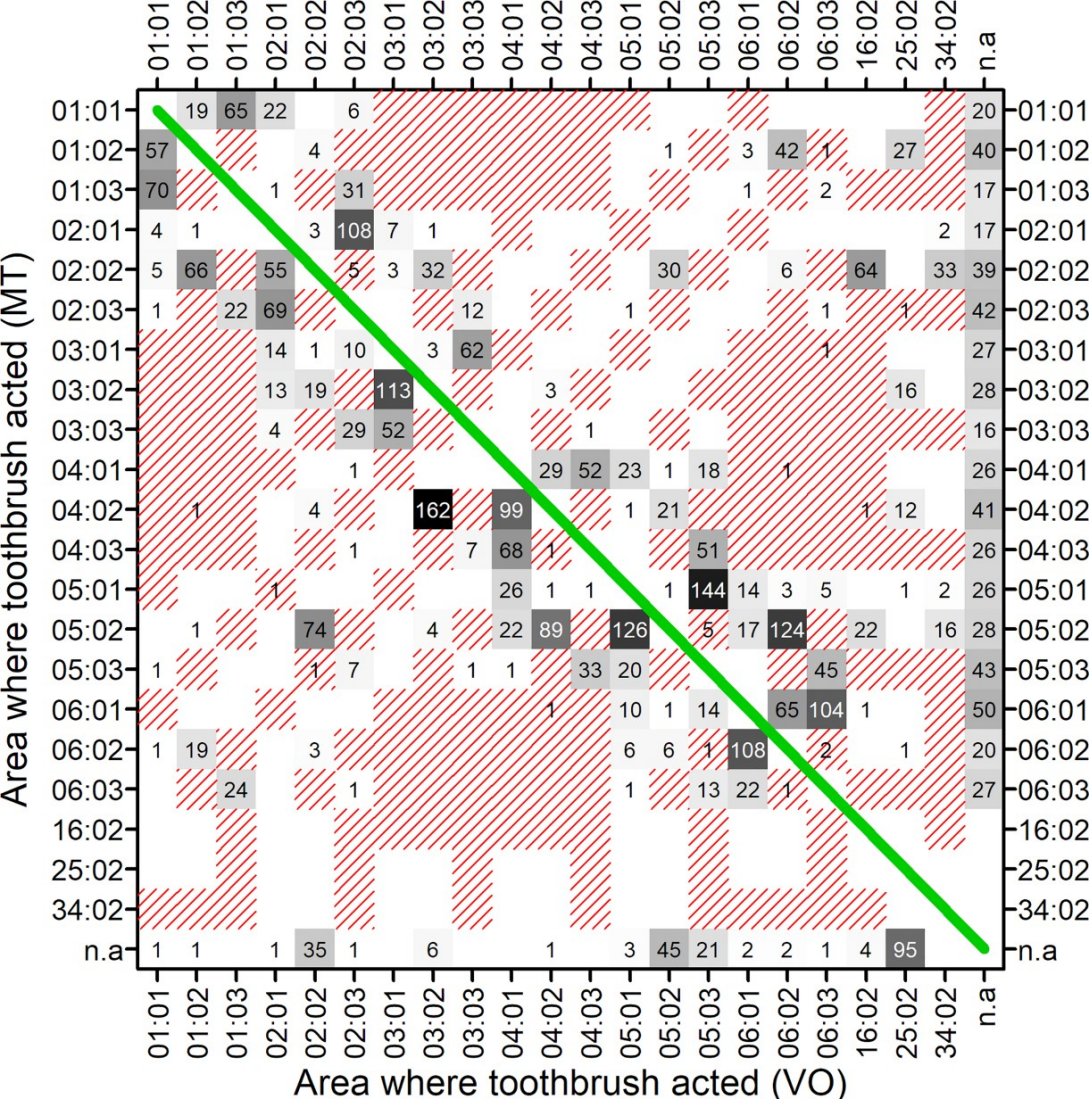
Heat map of confusion matrix based on first part of the study. The first pair of digits on the axes is the sextant (01–06; 16, 25 34 = subjects brushed opposing lateral right, frontal and lateral left sextants simultaneously with closed jaws), the second pair of digits is the surface (01 = occlusal/incisal, 02 = vestibular, 03 = oral). Correct classifications indicated by the diagonal line are excluded. Numbers and corresponding grey-shading indicate total numbers of mismatches out of the total of 22757 intervals considered (omitted if no respective mismatches, i.e. if equal to 0). Red-shaded areas indicate non-adjacent (i.e. more problematic) confusions, e.g. opposite sides of the mouth or vestibular vs. oral surface.

The heat map of the confusion matrix (Fig 2) shows for the first part of the study that most mismatches occurred with occlusal/incisal versus smooth surfaces (both oral and vestibular) and with neighbouring sextants. The pattern was the same when the data from both parts of the study were considered.

**Fig 3:**
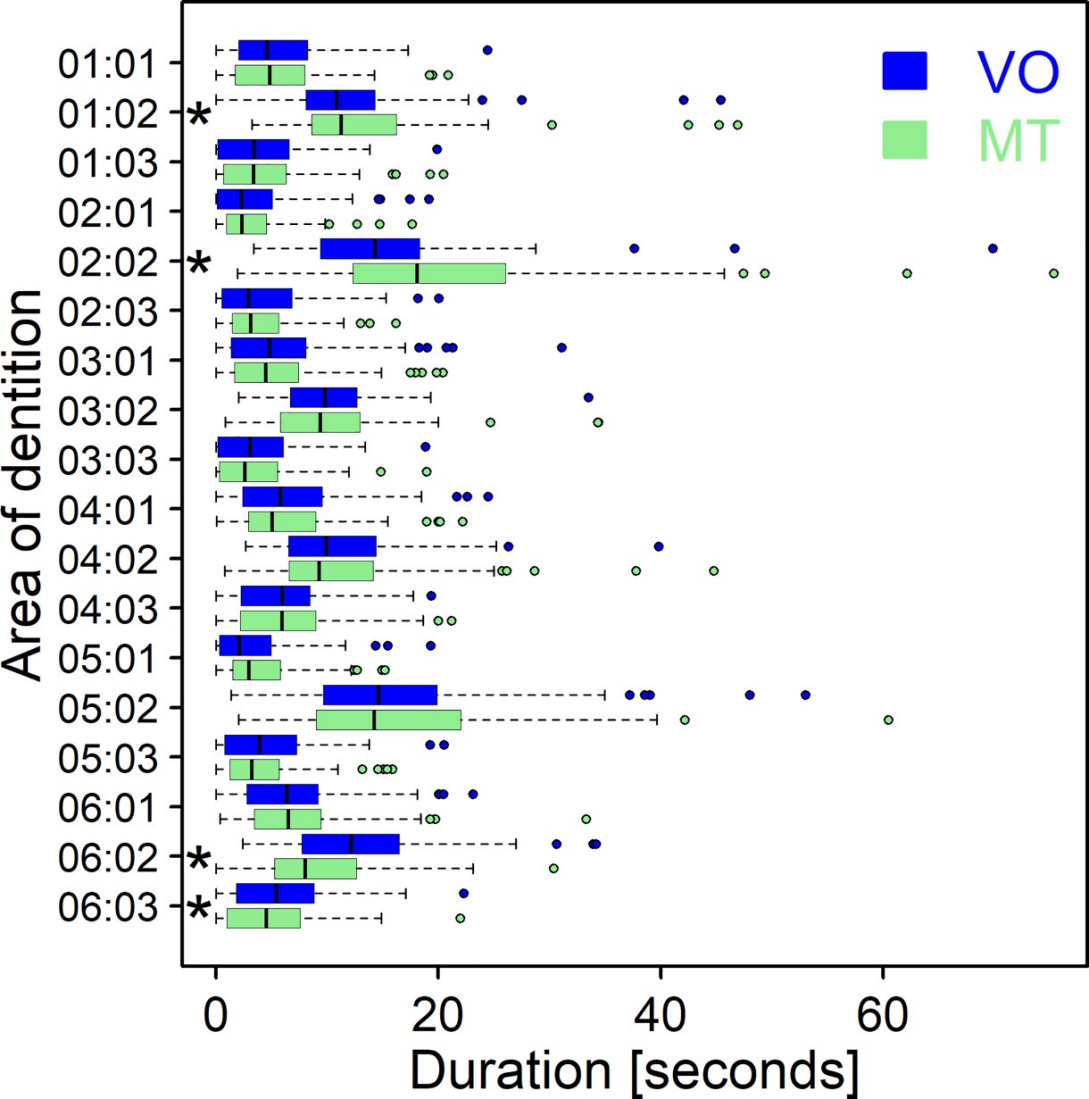
Boxplots of the duration of brushing in the different areas of the dentition. The first pair of digits is the sextant (01–06), the second pair of digits is the surface (01 = occlusal/incisal, 02 = vestibular, 03 = oral). Asterisks indicate significant differences between VO and MT at level 5%.

The duration (s) of brushing in the different areas is shown in Fig 3. VO and MT revealed very similar results with no significant difference except for 01:02 (10.8 (0.0; 45.4) versus 11.3 (3.2; 46.9); p_corrected_ = 0.008), 02:02 (14.3 (3.4; 69.9) versus 18.1 (1.9; 75.3); p_corrected_ < 0.001), 06:02 (12.2 (2.4; 34.2) versus 8.1 (0.0; 30.4); p_corrected_ < 0.001) and 06:03 (5.5 (0.0; 22.3) versus 4.6 (0.0; 22.0); p_corrected_ < 0.001).

### Toothbrushing systematics

For the first part of the study, data for parameters of interest are shown in Table 2.

**Table 2:** Parameters of systematics obtained from video observation (VO) and motion tracking (MT) for manual (M) and powered (P) toothbrushing, first part of the study only.

		TSI	Isochronicity	Consistency	Events
M	VO	1.26 (0.70; 1.70)	0.74 (0.48; 0.91)	0.58 (0.20; 0.86)	28.0 (11; 89)
	MT	0.80 (0.45; 1.52)	0.71 (0.36; 0.88)	0.21 (0.00; 0.71)	71.0 (24; 287)
P	VO	1.34 (0.59; 1.71)	0.75 (0.45; 0.93)	0.60 (0.01; 0.93)	35.0 (10; 113)
	MT	1.24 (0.64; 1.67)	0.71 (0.39; 0.85)	0.57 (0.00; 0.83)	42.5 (19; 128)

Data are given as median (min; max), all comparisons of VO and MT: p < 0.001

The number of events was distinctly higher when obtained from MT, which can be attributed to the higher resolution of the data recording (i.e. 100Hz, whereas in VO, only events of at least about 0.5 seconds duration are identified as individual events), but there was a significant correlation to values obtained from VO (manual toothbrush (n = 51): *ρ* = 0.52, p < 0.001; powered toothbrush (n = 52): *ρ* = 0.87; p < 0.001). Correspondingly, the TSI was significantly lower when calculated from MT data compared to VO. This is mainly due to consistency, which considers the completeness of reached areas as well as the number of events. The higher the number of events is, the lower is the value for consistency. The high number of events retrieved from MT is therefore related to the lower value for consistency. The other part of the TSI is isochronicity, which describes how evenly the brushing duration is distributed amongst the areas of the dentition. Despite the brushing duration in the various areas being quite similar between VO and MT, the isochronicity values obtained from the two observation methods were statistically significantly different, likely driven by the differences shown in Fig 3. Either way, the magnitude of the differences is deemed to be of minor clinical relevance.

The TSI increased significantly after instruction for both toothbrush types and observation methods (Table 3).

**Table 3:** Parameters of systematics obtained from video observation (VO) and motion tracking (MT) for manual (M) and powered (P) toothbrushing; b = baseline, pi = post instruction, d = difference post instruction–baseline.

			TSI	Isochronicity	Consistency	Events
M	VO	b	1.42 (0.70; 1.70)	0.76 (0.48; 0.91)	0.67 (0.22; 0.86)	25.5 (11; 89)
		pi	1.60 (1.09; 1.77)	0.84 (0.65; 0.92)	0.75 (0.44; 0.92)	13.5 (9; 73)
		d	0.18 (-0.03; 0.87)	0.07 (-0.02; 0.36)	0.09 (-0.05; 0.51)	-13.0 (-36; 9)
	MT	b	0.93 (0.50; 1.52)	0.72 (0.43; 0.88)	0.24 (0.00; 0.71)	69.5 (24; 246)
		pi	1.34 (0.71; 1.69)	0.82 (0.38; 0.92)	0.53 (0.06; 0.85)	29.5 (12; 103)
		d	0.33 (-0.03; 0.90)	0.09 (-0.04; 0.40)	0.25 (-0.15; 0.58)	-36.0 (-153; 10)
P	VO	b	1.38 (0.66; 1.71)	0.76 (0.54; 0.87)	0.60 (0.01; 0.93)	28.5 (10; 94)
		pi	1.63 (1.20; 1.81)	0.85 (0.65; 0.91)	0.77 (0.55; 0.93)	13.0 (8; 35)
		d	0.21 (-0.05; 0.97)	0.07 (-0.06; 0.36)	0.16 (-0.04; 0.76)	-13.5 (-76; 5)
	MT	b	1.24 (0.65; 1.67)	0.75 (0.50; 0.85)	0.55 (0.00; 0.83)	40.0 (19; 91)
		pi	1.52 (1.07; 1.74)	0.80 (0.64; 0.89)	0.72 (0.33; 0.85)	19.0 (11; 63)
		d	0.22 (-0.15; 0.99)	0.08 (-0.06; 0.27)	0.12 (-0.08; 0.78)	-15.5 (-74; 22)

Data are given as median (min; max), and d based on differences within subject. All differences between baseline and post instruction were significant with p ≤ 0.001.

## Discussion

The present study is the first comprehensive evaluation of a MT system as a tool for observing toothbrushing behaviour. Requirements for an alternative method to the visual observation are being able to measure a broad range of habitual behaviours relevant for oral hygiene and being able to measure behaviour changes after an intervention. Further, the method should be applicable to different types of toothbrushes. To test these aspects, we investigated not only habitual toothbrushing with both manual and powered toothbrushes, but included also a subgroup of subjects, who were instructed how to brush their teeth in a systematic way and were subsequently observed a second time.

The gold standard for comparison was the recording of toothbrushing behaviour by video followed by the visual analysis supported by a special software, which is a tool for comprehensive observational analysis. Observations on videos have obvious advantages over life observer coding and have been used in the field of oral hygiene already in the seventies [6–8, 25]. The parameters of interest used here were derived in part from these studies and covered position of the toothbrush per time point, the overall brushing time, the brushing time on areas of the dentition, and the number of changes between areas of the dentition. In addition, the degree of toothbrushing systematics was measured with the TSI Index [21]. In this index, toothbrushing is considered as systematic when all areas of the dentition are reached, the brushing time is equally distributed between all areas and when frequent alternations between areas are avoided. The motion tracking system used here included trackers mounted on the toothbrushes and one could assume that these devices have an influence on brushing behaviour. This aspect is of minor importance here but could be relevant for further applications of the system. Therefore, despite the time spent in the different sextants not varying much when participants brushed with or without trackers, miniaturisation of the system would be desirable.

The group of subjects included was a convenience sample of 103 middle-aged adults enrolled from regional dental practices, and it could be expected that this group would represent a broad range of toothbrushing behaviour.

Indeed, the video observation revealed that the included group presented the typical toothbrushing behaviour as observed earlier [6–8, 11, 22]. This behaviour is characterised by moving frequently between areas of the dentition and brushing predominantly on buccal surfaces while neglecting the oral surfaces. In addition, toothbrushing is often incomplete, meaning that not all areas of the dentition are reached. This was the case for both brushing with manual and powered toothbrushes, thereby confirming earlier results [22].

This behaviour changed after watching the instruction video, when all parameters of toothbrushing systematics improved. The number of events decreased distinctly, meaning that subjects brushed in a more consistent way, i.e. staying longer in an area instead of switching frequently from the right to the left. Further, the time spent on oral and vestibular surfaces was much more balanced compared to baseline.

Motion tracking was able to mirror the observations from video analysis. The median proportion of matches for areas (surface as well as sextant concurrent for both methods) was around 88%, which is in the order of the inter- and intra-rater agreement for VO. Correspondingly, there were no significant differences for brushing duration in the different areas with a few exceptions of minor order. Mismatches occurred mostly in neighbouring areas, i.e. between smooth surfaces and occlusal surfaces and between neighbouring sextants. In particular, this was the case when subjects frequently brushed in the border regions of areas. Brushing in border regions was often allocated to only one sextant/surface by VO while MT identified a change between the neighboured sextants.

Observing motor behaviour by a human observer on a two-dimensional screen is substantially different from tracking movements of an object in a three-dimensional space by a machine. While human observation can react easily to varying situations, machine observation requires fixed settings and algorithms. This was a minor problem for identifying the sextant where the brush acted, but a significant challenge for discriminating occlusal from smooth surface brushing. For VO, the surface where the majority of the toothbrush bristles was acting was coded. This, however, was a parameter, which was not suitable for MT. Therefore, preliminary tests were run to find a threshold angle of the toothbrush relative to the dental arc, which fits best to VO observations. Finally, it turned out that an angle of 20° of the toothbrush relative to the dentition was suitable for discriminating oral and vestibular from occlusal brushing on the molars. For front teeth, an angle of 13° was found useful for discriminating oral and vestibular from incisal brushing. Exploring the robustness of the results, it was found that changes in the angle of about 2–3° did not have a major impact overall, so modifications of the angles are not likely to offer substantial potential for further improvements. This is possibly due to variation in the thresholds applied by a human observer, whereas the threshold in MT is fixed and consistently applied. Still, as the distinction between smooth surface and occlusal brushing was still a frequent cause for mismatches, further efforts to refine the system may be helpful.

The video observation method used here was a controlled observation using standardised procedures, and the observer has coded the behaviour according to a previously defined scale. Though thorough calibration can improve the inter- and intra-rater-agreement, an inherent problem of human observation persists. If a subject observes, it can only do so within a professionalized discourse, which consists of traditional notions and an ideal model of explanation [26]. Thus, any observation is inherently an interpretative process. Against this background, it is clear that a professional observer will interpolate the action of a toothbrush in a meaningful way in cases of borderline locations whereas the machine follows rigid algorithms. Besides the fact that mismatches clustered at occlusal/smooth surfaces and neighboring sextants, the most striking difference between the both observation methods was the number of events, which was much higher from MT than from VO. In part, this can be explained by the high time resolution of the motion tracking system, but the difference persisted even after aggregating the MT and VO data to comparable intervals. An additional impact might arise from spatial imprecision of the VO coding: e.g. in case of slow brushing on the boundary between areas, a human observer might rather interpret the pattern and decide not to code a new event at each crossing of the boundary, while MT will strictly assign the position to the corresponding area. As a result of the higher rate of events, the TSI obtained from MT was much lower than from VO. This was mainly due to the consistency calculation where the number of events is included. Nevertheless, motion tracking was able to demonstrate improved toothbrushing systematic in a similar way.

Besides location and sequence of toothbrushing, the brushing technique has been regarded in video observations and is part of oral hygiene instructions. For adults, the modified Bass technique is the most commonly recommended technique but sometimes also circling or scrub techniques were suggested [27]. So far, none of the diversity of brushing techniques seems to be systematically superior to others, thus the significance of adopting a specific brushing technique remains unclear. Even if toothbrushing systematics surely is much more important than the brushing technique, it would be desirable to identify brushing techniques with MT. Video observations have shown that subjects do not brush with the same technique throughout the dentition; instead, many different movements are performed. As mentioned above, it is easy for a professional observer to categorize this diversity of movements into classes like circling, horizontal, vertical or jiggling, but this is much more difficult to translate into algorithms for motion tracking. In this context, machine learning was used to distinguish a toothbrushing behavior which was defined as correct from all other toothbrushing behaviors [15]. This, however, is only a simplified first step towards capturing the complex and highly variable gestures of habitual toothbrushing, which is surely a field for future research.

## Conclusions

VO and MT are very different methods of operation for recording and analysing toothbrushing behaviour. Despite this, MT was able to provide well-matching data with VO for position of the toothbrush per time point, the overall brushing time, and the brushing time on areas of the dentition. Thus, we can conclude that motion tracking is a suitable tool to locate the toothbrush during toothbrushing relative to the dentition regardless of the toothbrush type (powered or manual). Future applications could include the objective measurement of the degree of toothbrushing systematics as well as of improvements of toothbrushing behaviour after instruction.
